# Gemini Alkyldeoxy-D-Glucitolammonium Salts as Modern Surfactants and Microbiocides: Synthesis, Antimicrobial and Surface Activity, Biodegradation

**DOI:** 10.1371/journal.pone.0084936

**Published:** 2014-01-08

**Authors:** Bogumił Brycki, Adrianna Szulc

**Affiliations:** Laboratory of Microbiocides Chemistry, Faculty of Chemistry, Adam Mickiewicz University, Poznań, Poland; University of Kansas, United States of America

## Abstract

Dimeric quaternary alkylammonium salts possess a favourable surface and antimicrobial activity. In this paper we describe synthesis, spectroscopic analysis, surface and antimicrobial activity as well as biodegradability of polymethylene-α,ω-bis(*N,N*-dialkyl-*N*-deoxy-D-glucitolammonium iodides), a new group of dimeric quaternary ammonium salts. This new group of gemini surfactants can be produced from chemicals which come from renewable sources. The structure of products has been determined by the FTIR and ^1^H and ^13^C NMR spectroscopy. The biodegradability, surface activity and antimicrobial efficacy against *Escherichia coli*, *Staphylococcus aureus*, *Candida albicans*, *Aspergillus niger* and *Penicillium chrysogenum* were determined. The influence of the number of alkyl chains and their lengths on surface and antimicrobial properties has been shown. In general, dimeric quaternary alkyldeoxy-D-glucitolammonium salts with long alkyl substituents show favourable surface properties and an excellent antimicrobial activity.

## Introduction

Surfactants have many different applications; not only as household chemicals, but they are also used in the petrochemical, mining, cosmetics and pharmaceutical industry. Some surfactants are also applied in the “hi-tech” fields, especially in nanotechnology, molecular biology and nanomedicine [1]–[3]. Due to the huge consumption of surfactants, over 10 millions tonnes per annum and the risk of pollution of environment, there is a strong demand to obtain new, more effective and environment friendly surfactants. Among the others, more effective surfactant means a surfactant which can be used in smaller amounts to give the same or better surface effect in comparison to a classical surfactant. Double quaternary alkylammonium salts -gemini surfactants- belong to this new class of more effective surfactants. Gemini surfactants possess at least two hydrophobic hydrocarbon chains and two hydrophilic quaternary ammonium groups, which are connected by a spacer. The spacer can be either hydrophobic (polymethylene chain), or hydrophilic (polymethylene chain with ether or hydroxyl groups). From a structural point of view a spacer can be rigid (aromatic or unsaturated linear hydrocarbons) or flexible (polymethylene chain). The neutral charge of the molecule is retained by the presence of counterions, which usually are halide anions [4]–[8]. The gemini alkylammonium salts show unique surface and interfacial properties in aqueous solution. Critical micelle concentrations (CMC) of gemini surfactants are usually much lower than CMC's of corresponding monomeric surfactants [3], [4], [8]–[13]. The gemini alkylammonium compounds show also a very good antimicrobial activity against bacteria, viruses, molds and yeasts [14], [15]. The minimal inhibitory concentrations (MIC) of these compounds in some cases are even three orders of magnitude lower in comparison to their monomeric analogs [9], [16], [17]. The mechanism of biocidal activity of quaternary alkylammonium salts is based on adsorption of the alkylammonium cation on the bacterial cell surface, diffusion through the cell wall and then binding and disruption of cytoplasmatic membrane. Damage of the membrane results in a release of potassium ions and other cytoplasmatic constituents, finally leading to the death of the cell [9], [18], [19]. A frequently used microbiocides, especially in sublethal concentrations, can imply an increasing resistance of microorganisms. One of the way to overcome this serious negative side effect is a periodically application of new microbiocides with modified structures. One of a new type of gemini surfactants with advantageous surface and antimicrobial activity are sugar based gemini surfactants [20]–[22]. Sugar based gemini surfactants have been also tested as vectors for gene transfer, which has a great importance in gene therapy [23]–[26]. Synthesis of some nonionic types of those compounds was previously described [27]–[30].

**Figure 1:**
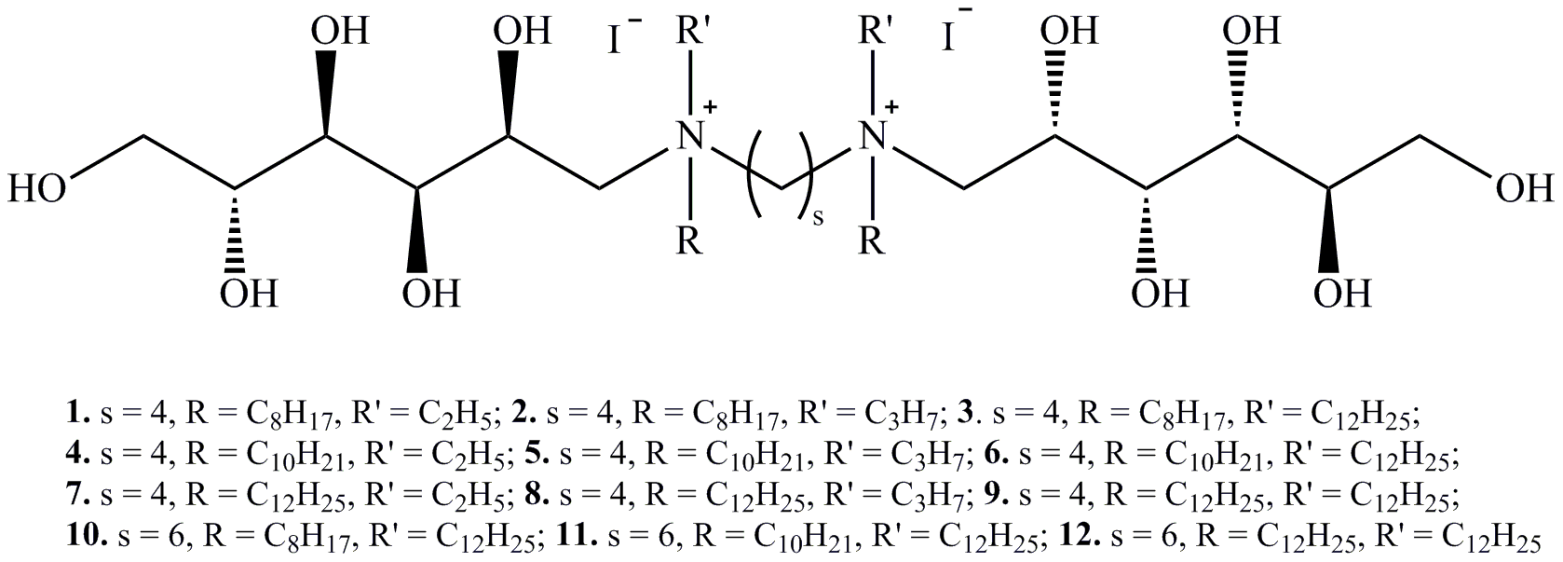
Structure of synthesized alkyldeoxy-D-glucitolammonium salts. Spacer (s) may have 4 or 6 methylene groups; hydrocarbon substituents (R, R′) can be ethyl, propyl, octyl, decyl or dodecyl group.

In this paper we report synthesis, spectroscopic analysis, surface and antimicrobial activity, as well as biodegradability of new cationic gemini alkylammonium surfactants with deoxy-D-glucitol substituents (Figure 1) and their precursors. The synthesis, crystal structure, FTIR, NMR and B3LYP results of a bolaform molecule has been previously reported [31].

## Materials and Methods

### Materials

D-glucose, tetramethylenediamine, hexamethylenediamine, iodoethane, 1-iodopropane, 1-iodododecane were purchased from Sigma-Aldrich. Hexanal was obtained from Fluka. Octanal, decanal, dodecanal, sodium borohydride and sodium cyanoborohydride were purchased from Merck. Sodium hydroxide, potassium hydroxide, hydrochloric acid, acetic acid, methanol, 2-propanol, *N,N*-dimethylformamide and diethyl ether were obtained from Chempur. Nitromethane was purchased from POCH. All reagents and solvents were used as obtained without further purification.

### Measurements

Melting points were determined using Electrothermal Met-Temp apparatus. Elemental analysis were performed on Elemental Analyser Vario EL II. NMR spectra were recorded on a Varian VNMR-S spectrometer operating at 402.644 MHz and 101.244 MHz for ^1^H and ^13^C respectively. The spectra were measured in D_2_O solutions relative to internal standard 3-(trimethylsilyl)propionic-2,2,3,3-d_4_ acid sodium salt, or in TFA-d solutions relative to internal standard tetramethylsilane. The 2D ^1^H-^1^H (COSY), ^1^H-^13^C (HETCOR) spectra were performed on Bruker Avance 600 MHz. The FTIR spectra were recorded in KBr pellets on a Bruker IFS 66 v/S spectrometer, evacuated to avoid water and CO_2_ absorptions, at 2 cm^−1^ resolution. The electron spray ionization mass spectra (ESI-MS) were recorded on ZQ Waters mass spectrometer. The sample solutions were prepared in methanol or water. The ESI-MS spectra were recorded at a 30 V cone voltage.

Antimicrobial activity of the synthesized compounds was evaluated against two species of bacteria: *Escherichia coli* ATCC 10536 and *Staphylococcus aureus* ATCC 6538, yeast: *Candida albicans* ATCC 10231, and two species of mold: *Aspergillus niger* ATCC 16401 and *Penicillium chrysogenum* ATCC 60739. Minimal inhibitory concentrations (MIC) were measured by a tube standard 2-fold dilution method, i.e. the volume of the original solution is always doubled, as in going from 1 to 2. Bacteria were preincubated on Tripticase Soy Broth (TSB) slant for 1 day at 37°C and fungi were preincubated on Malt Extract Broth (MEB); mold for 5 days at 28°C, yeast – for 1 day at 37°C. Conidia suspensions were prepared by adding sterile water containing 0.1% (w/w) Tween 80 to the slant. The bacteria and yeasts cell suspension were prepared by similar procedure but without Tween 80. One mL of inoculum (density 10^6^ cells mL^−1^) was mixed with 1 mL of media containing the tested compounds and incubated for 24 h at 30°C for fungi, and 37°C for bacteria. The MIC's were defined as the lowest concentrations of the compounds at which there was no visible growth.

Surface tension measurements were performed on Krüss 100 KC tensiometer with Dosimat control. Critical micelle concentrations (CMC) were determinated by the cross-point of the lines before and after CMC on the surface tension vs. log[concentration of surfactants] curve.

Biodegradability of the synthesized surfactants was examined using a dissolved organic carbon (DOC) die-away test according to OECD guideline 301A [32]. The inoculum was obtained from activated sludge from wastewater treatment plant. To the flask was added 250 mL of medium and adequate amount of test compound (to obtain concentration DOC in a range 10–40 mg L^−1^). Sequentially inoculated with activated sludge, to the concentration of dry weight was lower than 30 mg L^−1^ and supplemented by a medium to volume 500 mL. Blank sample was prepared in the same way, but without tested compound. Incubation was carried out in the indirect light at temperature 20–25°C. Test was conducted in the incubator. Aerating the solutions were obtained by the shaker. Biodegradation of the test substance was monitored by determination of DOC dye-away and was calculated using the following formula: 
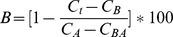
where:

B is percentage of biodegradation of the test compound, %

C_t_ is average value of DOC after time t, mg L^−1^

C_B_ is average value of DOC in blank sample after time t, mg L^−1^

C_A_ is average value of DOC after time 3 h±30 min, mg L^−1^

C_BA_ is average value of DOC in blank sample after time 3 h±30 min, mg L^−1^.

### Synthesis

**Figure 2:**
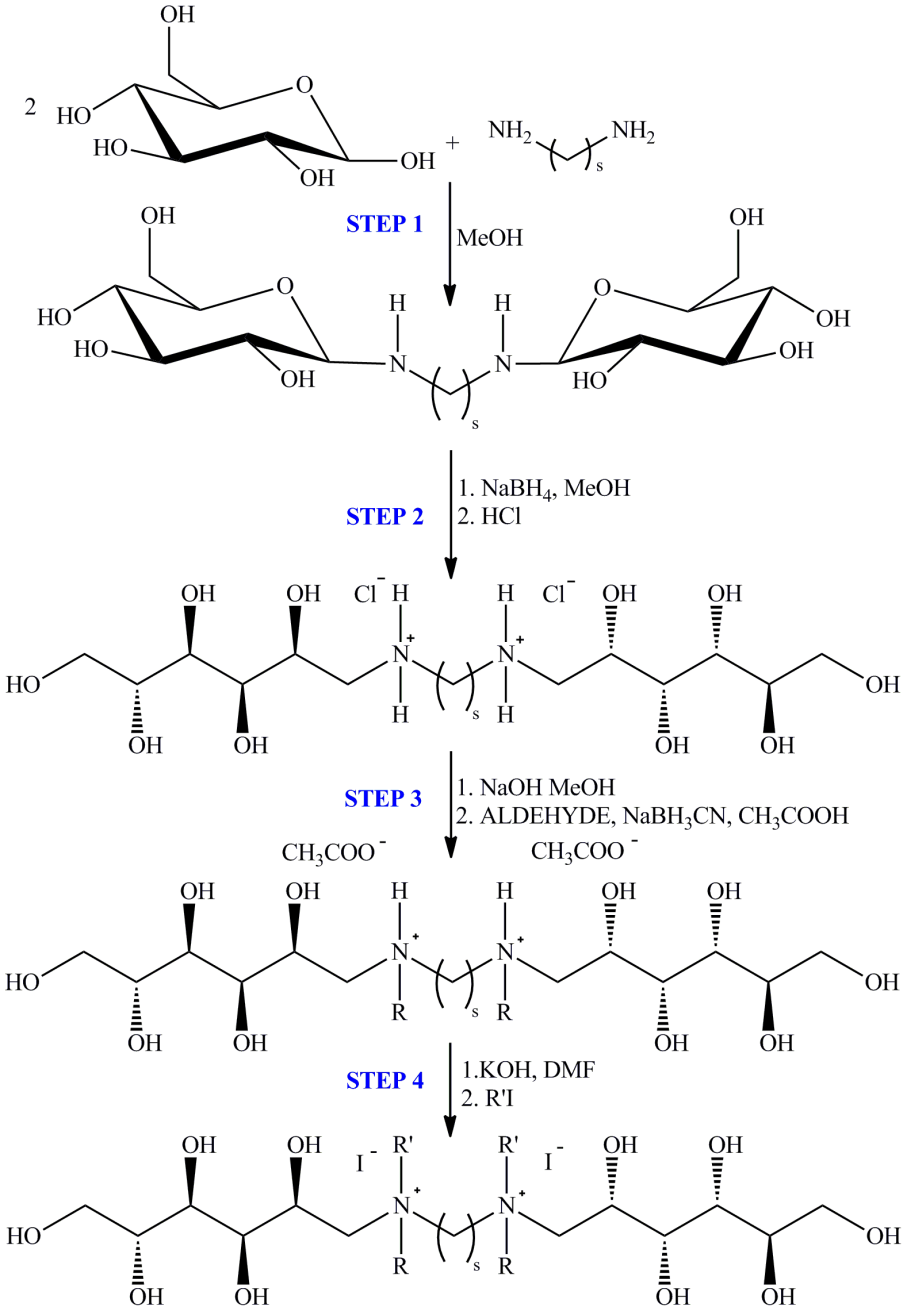
Four-steps synthesis of polymethylene-α,ω-bis(*N,N*-dialkyl-*N*-deoxy-D-glucitolammonium iodides). Condensation of D-glucose and diamine (Step 1, reduction of D-glucopyranosyl rings to deoxy-D-glucitol substituents (Step 2), reductive alkylation with aldehydes (Step 3) and quaternization of nitrogen atoms by aliphatic iodides (Step 4).

Polymethylene-α,ω-bis(*N,N*-dialkyl-*N*-deoxy-D-glucitolammonium iodides) (**1–12**) were obtained in four-step synthesis (Figure 2). The first step, a condensation of D-glucose and diamine, gave products with glycosidic bonds. Step two, reduction of D-glucopyranosyle ring with sodium borohydride to deoxy-D-glucitol form, was followed by step 3, i.e. a reductive alkylation with aliphatic aldehydes, containing from 6 to 12 carbon atoms, in the presence of sodium cyanoborohydride as a selective reducing agent [33]. Quaternisation of nitrogen atoms by aliphatic *n*-iodides was the last, step 4 of the reaction procedure. Nonionic tetramethylene-1,4-bis(*N*-dodecyl-*N*-deoxy-D-glucitolamine) (**I-13**) (Figure S5) was also obtained for comparative study. The details of the syntheses are given in Materials and Methods S1.

## Results and Discussion

### Antimicrobial properties

Selected compounds were tested for antimicrobial activity against *Escherichia coli* ATCC 10536, *Staphylococcus aureus* ATCC 6538, *Candida albicans* ATCC 10231, *Aspergillus niger* ATCC 16401 and *Penicillium chrysogenum* ATCC 60739. The microorganisms used in our experiment are considered to be ubiquitous and commonly existing in hospitals and food industry plants. Minimum inhibitory concentration (MIC) values were determined and are given in Table 1.

**Table 1:** Antibacterial and antifungal activities of gemini alkyldeoxy-D-glucitolammonium salts as MIC's [µmol L^−1^].

Compound	*S. aureus*	*E. coli*	*C. albicans*	*A. niger*	*P. chrysogenum*
**1**	31.6	31.6	31.6	>127	31.6
**2**	31.6	31.6	31.6	>127	31.6
**3**	20	20	20	20	20
**4**	31	25	31	31	31
**5**	30	30	30	30	30
**6**	24	19	24	9.7	9.7
**7**	29	29	3.8	7.5	7.5
**8**	28	28	1.8	7.3	7.3
**9**	12	19	23	23	12
**10**	20	20	20	20	20
**11**	19	19	19	19	19
**12**	18	18	18	9.1	9.1

The MIC data presented in Table 1 show a distinct relationship between structure and antimicrobial activity of dimeric alkylammonium surfactants. Compounds with no hydrocarbon substituents at nitrogen atom, tetramethylene-1,4-bis(*N*-deoxy-D-glucitolammonium chloride) (**I-3**) (Figure S2) and hexamethylene-1,6-bis(*N*-deoxy-D-glucitolammonium chloride) (**I-4**) (Figure S2), show a very weak antimicrobial activity, with MIC over 240 µmol L^−1^. Similarly, MIC value for nonionic tetramethylene-1,4-bis(*N*-dodecyl-*N*-deoxy-D-glucitolamine) (**I-13**) (Figure S5), where nitrogen atoms are not charged, is over 240 µmol L^−1^. All synthesized gemini surfactants with long alkyl and deoxy-D-glucitol substituents (compounds **1–12**) show a very good antimicrobial activity against Gram positive bacteria, Gram negative bacteria, and fungi. Dimeric quaternary alkyldeoxy-D-glucitolammonium salts with tetramethylene spacer show a better biocidal efficacy against microorganisms in comparison to compounds with the longer, hexamethylene spacer. Moreover, the highest antifungal activity was observed for quaternary ammonium derivatives, which contain one long and one short hydrocarbon chain (compounds **7** and **8**). In both these cases, the ionic radius is smaller, in comparison to deoxy-D-glucitolammonium salts with hexamethylene spacer or deoxy-D-glucitolamonium salts with two long alkyl chains. The smaller ionic radius of cation causes stronger Coulombic interactions with the negatively charged cell surface of microorganisms and as a consequence the higher biocidal activity. MIC values of polymethylene-α,ω-bis(*N,N*-dialkyl-*N*-deoxy-D-glucitolammonium iodides) are lower than minimal inhibitory concentrations of conventional gemini alkylammonium surfactants with [n-s-n] structure, where “n” is a number of carbon atoms in the hydrocarbon chain and “s” is a number of methylene groups in the spacer [14], [16]. This phenomenon is a result of the presence of deoxy-D-glucitol substituents in the molecule, which allow more efficient adsorption of the ammonium cation on the microorganism cell surface. The very high antimicrobial activity of dimeric quaternary alkyldeoxy-D-glucitolammonium salts has a fundamental meaning from ecological point of view. These compounds show the same biocidal effectiveness like conventional quaternary ammonium salts or other biocides but in hundred times smaller amounts. Therefore their environmental fate is much smaller [34].

### Surface properties

Most gemini alkylammonium salts are typical surfactants. The surface activity of these compounds is much higher than for corresponding monomeric surfactants. This efficiency is often characterized either by the concentration C_20_, i.e. the surfactant concentration required to lower the surface tension of water by 0.02 N/m, or critical micelization concentration, CMC. For typical gemini surfactant, ethylene-1,2-bis(*N*-dodecyl-*N,N*-dimethylammonium bromide) [12-2-12], CMC value is 0.82 mmol L^−1^ and surface tension at the critical micelle concentration is 30.6 mN m^−1^ [11] while for dodecyltrimethylammonium bromide (DTAB) these values are 15.1 mmol L^−1^ and 36.4 mN m^−1^, respectively [11]. Similarly, the values of C_20_ for 12-2-12 and DTAB are 0.0083 and 0.21 wt%, respectively [4]. The reason for this greater surface activity of gemini surfactants in the comparison to analogues monomeric surfactants, is the larger total number of carbon atoms in the hydrophobic chains of the geminis. The larger the total number of carbon atoms in the surfactant molecule, the greater the distortion of the water structure of the aqueous phase and the greater the tendency to adsorb at the interfaces surrounding the aqueous phase or to form micelles in the aqueous phase, that is, greater surface activity [3]. Surface tension at CMC (γ_CMC_) and critical micelle concentrations (CMC) of dimeric quaternary alkyldeoxy-D-glucitolammonium salts are given in Table 2. Dimeric alkyldeoxy-D-glucitolammonium iodides show an excellent surface activity because of their amphiphilic structure. The CMC values of investigated iodides are much lower than CMC's of gemini alkylammonium salts with analogous lengths of hydrocarbon chain, where instead of deoxy-D-glucitol substituent is methyl group. Dimeric alkyldeoxy-D-glucitolammonium salts **1–12** containing two hydrocarbon chains show lower CMC values than those with a single hydrocarbon chain. For example CMC values for tetramethylene-1,4-bis(*N*-dodecyl-*N*-deoxy-D-glucitolammonium acetate) (**I-8**) (Figure S3) and hexamethylene-1,6-bis(*N*-dodecyl-*N*-deoxy-D-glucitolammonium acetate) (**I-12**) (Figure S4) are 0.17 and 0.12 mmol L^−1^, respectively. It is important to note that one hydrocarbon chain must be long, at least 8 carbon atoms, however the second one can be shorter. Moreover, dimeric alkyldeoxy-D-glucitolammonium salts containing six methylene groups in the spacer show lower CMC values, than compounds with tetramethylene spacer. This is due to the morphology of micelles in the solution [35]. To form micelles, fewer of bigger cationic molecules are needed, i.e. critical micelle concentration is lower.

**Table 2:** Surface tension at CMC (γ_CMC_) and critical micelle concentrations (CMC) values of gemini alkyldeoxy-D-glucitolammonium surfactants.

Compound	γ_CMC_ [mN m^−1^]	CMC [mmol L^−1^]
**1**	28.5	0.03
**2**	29.3	0.03
**3**	25.9	0.01
**4**	26.5	0.03
**5**	28.5	0.04
**6**	25.4	0.009
**7**	29.3	0.06
**8**	28.5	0.07
**9**	26.0	0.01
**10**	28.1	0.01
**11**	32.5	0.03
**12**	27.2	0.04

### Biodegradability

Alkylammonium gemini surfactants are poorly biodegradable because of their biocidal properties as well as strong adhesive properties. Banno et al. have studied biodegradation of conventional gemini cationic surfactants [36], [37]. They have proved that the homologous C10, C12 and C14 of pentamethylene-1,5-bis-(*N*-*n*-alkyl-*N,N*-dimethylammonium iodide) and trimethylene-1,3-bis-(*N*-dodecyl-*N,N*-dimethylammonium iodide) showed practically no biodegradation by activated sludge. The biochemical oxygen demand (BOD) biodegradability for homologue C10 was only about 10%.

However modification of the structure or addition of natural origin substituent, can significantly affect biodegrability. Compounds containing easily hydrolyzed groups in the structure showed a good biodegradability [36]. Generally readily biodegradable compounds achieve 70% removal of dissolved organic carbon (DOC) and 60% of theoretical oxygen demand (ThOD) or CO_2_ production within 28 days [32].

Introduction of the carbonate linkage into the hydrophobic moiety of gemini surfactants enhanced its biodegradability. However, biodegradation of this surfactant was still low, only about 25% after 28 days of incubation for (C_12_H_25_-O-CO-O-(CH_2_)_3_-N^+^(CH_3_)_2_-(CH_2_)_3_-N^+^(CH_3_)_2_-(CH_2_)_3_-O-CO-O-C_12_H_25_ 2I^−^). The biodegradability of the primary biodegradation intermediates: 1-dodecanol and trimethylene-1,3-bis(*N*-3-hydroxypropyl-*N*,*N*-dimethylammonium iodide) were about 70% and 10% respectively. Based on these results, one can state that the low biodegradability of studied gemini surfactant are due to the low biodegradability of the intermediate having two ammonium groups [36], [37]. Introduction of the carbonate linkage into the linker moiety between the two single-type cationics, or in both the hydrophobic and linker moieties, significantly improved the biodegrability of those surfactants. The maximum BOD-biodegradability of homologous C12 exceeded 70% after a 28-day incubation [36], [37]. A comparison of the biodegradability of surfactants with two and three methylene groups in the linker containing carbonate group showed much higher degree of biodegradation for the former compounds. It is due to electron density of the carbonyl carbon. For compound with two methylene groups the carbonyl carbon atom is more influenced by charged nitrogen atom in comparison to compound with three methylene groups in the linker [36], [37].

In our study, we performed biodegradability measurements for different compounds: neutral dimeric surfactant tetramethylene-1,4-bis(*N*-dodecyl-*N*-deoxy-D-glucitolamine) (**I-13**) (Figure S5), the complex of dimeric surfactant with the weak hydrogen bond, hexamethylene-1,6-bis(*N*-dodecyl-*N*-deoxy-D-glucitolammonium acetate) (**I-12**) (Figure S4), protonated dimeric surfactants tetramethylene-1,4-bis(*N*-deoxy-D-glucitolammonium chloride) (**I-3**) (Figure S2) and hexamethylene-1,6-bis(*N*-deoxy-D-glucitolammonium chloride) (**I-4**) (Figure S2), as well as quaternized dimeric surfactants **1–12**. The nonionic compound, tetramethylene-1,4-bis(*N*-dodecyl-*N*-deoxy-D-glucitolamine) (**I-13**) (Figure S5), undergoes the biodegradation up to 89.6±1.6%. The above compound is not charged, and no charged intermediates are formed during degradation process. Thus according to OECD is readily biodegradable [32]. In the case of the complex of dimeric surfactant with acetic acid, i.e. hexamethylene-1,6-bis(*N*-dodecyl-*N*-deoxy-D-glucitolammonium acetate) (**I-12**) (Figure S4), a weak hydrogen bond N…H-O exists between nitrogen atoms of surfactant and acetic acid, and nitrogen atoms are protonated to some extension. As a result of this partially protonated nitrogen atoms, the biodegradation is significantly lowered in comparison to biodegradation of neutral compound and equals 62.1±1.4%. In complexes of dimeric alkyldeoxy-D-glucitolamine with hydrochloric acid, i.e. tetramethylene-1,4-bis(*N*-deoxy-D-glucitolammonium chloride) (**I-3**) (Figure S2) and hexamethylene-1,6-bis(*N*-deoxy-D-glucitolammonium chloride) (**I-4**) (Figure S2), where nitrogen atoms are completely protonated, the biodegradation is very low and amounts 14.4±1.7% and 26.5±1.2%, respectively. The biodegradation data for dimeric quaternary alkyldeoxy-D-glucitolammonium iodides **1–12** are given in Table 3. These compounds are not easily degradated; the biodegradation degree does not exceed 34%.

**Table 3:** Biodegradation of gemini alkyldeoxy-D-glucitolammonium salts determined by DOC die-away test.

Compound	Biodegradation [%]
**1**	32.5±1.5
**2**	27.4±1.6
**3**	28.4±1.4
**4**	31.5±1.6
**5**	30.8±1.4
**6**	27.3±1.4
**7**	30.2±1.6
**8**	23.4±1.7
**9**	22.8±1.6
**10**	25.4±1.5
**11**	23.2±1.3
**12**	20.5±1.6

These results demonstrate that dimeric quaternary alkyldeoxy-D-glucitolammonium iodides **1–12** and completely protonated compounds, tetramethylene-1,4-bis(*N*-deoxy-D-glucitolammonium chloride) (**I-3**) (Figure S2) and hexamethylene-1,6-bis(*N*-deoxy-D-glucitolammonium chloride) (**I-4**) (Figure S2), are relatively resistant to the biodegradation. This is due to positively charged nitrogen atoms and no sensitive groups in the structure of these compound to be easily hydrolized. In the case of neutral tetramethylene-1,4-bis(*N*-dodecyl-*N*-deoxy-D-glucitolamine) (**I-13**) (Figure S5) and compound with weak hydrogen bonds, hexamethylene-1,6-bis(*N*-dodecyl-*N*-deoxy-D-glucitolammonium acetate) (**I-12**) (Figure S4) the degree of biodegradation is much higher. It clearly indicates that the charge on nitrogen atom play a crucial role in the biodegradation process of studied alkylammonium salts. These results are in good accordance with work of Banno and proposed mechanism of biodegradation of gemini surfactants [36], [37]. It is important to note that there is no simple correlation between antimicrobial activity and biodegradation. The high antimicrobial activity of the biocide against specific strains do not always correspond to its low biodegradability. The biodegradation of cationic surfactants requires the concerted action of at least two microorganisms because a single organism usually lacks the full complement of enzymatic capabilities. The mechanisms for the enhanced biodegradation of mixed cultures are the provision of specific nutrients, removal of growth-inhibiting products, and the combined metabolic attack on the substrate. Consortia are required for the degradation of not only cationic surfactants [38].

## Conclusions

A series of new gemini alkyldeoxy-D-glucitolammonium salts with tetramethylene and hexamethylene spacers and different hydrocarbon substituents have been synthesized and characterized by ^1^H NMR, ^13^C NMR, FTIR, ESIMS and elemental analysis. Tetramethylene-1,4-bis(*N*-alkyl-*N*-deoxy-D-glucitolammonium acetates) and hexamethylene-1,6-bis(*N*-alkyl-*N*-deoxy-D-glucitolammonium acetates) as intermediates have also been described. The obtained cationic sugar based gemini surfactants exhibit a high antimicrobial activity against bacteria and fungi. The biocidal efficacy depends on structure of gemini surfactant. The most active are compounds with two hydrocarbon substituents; one of them should possess at least eight methylene groups while the second one can be shorter. Compounds without hydrocarbon substituents show no biocidal activity. Minimal inhibitory concentrations of sugar based gemini surfactants are much lower in comparison both to gemini surfactants with no deoxy-D-glucitol substituents as well as monomeric alkylammonium salts.

Investigated dimeric alkyldeoxy-D-glucitolammonium salts show an excellent surface activity. CMC values are much lower then corresponding gemini surfactants with no sugar substituent. This is due to the morphology of micelles in the solution. In order to form micelle, fewer of bigger cationic molecules are needed and critical micelle concentration is lower.

The biodegradability of gemini alkyldeoxy-D-glucitolammonium salts is poor in comparison to neutral substrates. This is caused by positively charged nitrogen atoms and lack of sensitive groups in the structure of these compound to be easily hydrolized.

## Supporting Information

Figure S1 **Structure of polymethylene-1,n-bis(*N*-D-glucopyranosylamines). I-1** s = 4; **I-2** s = 6.
(TIF)

Figure S2 **Structure of polymethylene-1,n-bis(** ***N*** **-deoxy-D-glucitolammonium chlorides).** **I-3** s = 4; **I-4** s = 6.
(TIF)

Figure S3 **Structure of tetramethylene-1,4-bis(** ***N*** **-alkyl-** ***N*** **-deoxy-D-glucitolammonium acetates).** **I-5** R = C_6_H_13_; **I-6** R = C_8_H_17_; **I-7** R = C_10_H_21_; **I-8** R = C_12_H_25_.
(TIF)

Figure S4 **Structure of hexamethylene-1,6-bis(** ***N*** **-alkyl-** ***N*** **-deoxy-D-glucitolammonium acetates).** **I-9** R = C_6_H_13_; **I-10** R = C_8_H_17_; **I-11** R = C_10_H_21_; **I-12** R = C_12_H_25_.
(TIF)

Figure S5 **Structure of tetramethylene-1,4-bis(** ***N*** **-dodecyl-** ***N*** **-deoxy-D-glucitolamine) I-13.**
(TIF)

Figure S6 **Structure of tetramethylene-1,4-bis(** ***N*** **-alkyl-** ***N*** **-octyl-** ***N*** **-deoxy-D-glucitolammonium iodides).** **1** R′ = C_2_H_5_; **2** R′ = C_3_H_7_; **3** R′ = C_12_H_25_.
(TIF)

Figure S7 **Structure of tetramethylene-1,4-bis(** ***N*** **-alkyl-** ***N*** **-decyl-** ***N*** **-deoxy-D-glucitolammonium iodides).** **4** R′ = C_2_H_5_; **5** R′ = C_3_H_7_; **6** R′ = C_12_H_25_.
(TIF)

Figure S8 **Structure of tetramethylene-1,4-bis(** ***N*** **-alkyl-** ***N*** **-dodecyl-** ***N*** **-deoxy-D-glucitolammonium iodides).** **7** R′ = C_2_H_5_; **8** R′ = C_3_H_7_; **9** R′ = C_12_H_25_.
(TIF)

Figure S9 **Structure of hexamethylene-1,6-bis(** ***N*** **-dodecyl-** ***N*** **-alkyl-** ***N*** **-deoxy-D-glucitolammonium iodides).** **10** R = C_8_H_17_; **11** R = C_10_H_21_; **12** R = C_12_H_25_.
(TIF)

Materials and Methods S1 (DOC)
