# Supplementary material for: Gemini Alkyldeoxy-D-Glucitolammonium Salts as Modern Surfactants and Microbiocides: Synthesis, Antimicrobial and Surface Activity, Biodegradation
Source: PLoS One. 2014 Jan 8;9(1):e84936. doi: 10.1371/journal.pone.0084936 (PMC3885657; doi:10.1371/journal.pone.0084936)
Supplement: Materials and Methods S1 — (DOC) [file pone.0084936.s010.doc]

**Supplementary Materials and Methods**

**Tetramethylene-1,4-bis(*N*–D-glucopyranosylamine) (I-1)** (Figure S1) (IUPAC name: (2S,3R,4R,5S,6S)-2-(hydroxymethyl)-6-((4-(((2R,3R,4S,5S,6R)-3,4,5-trihydroxy-6-(hydroxymethyl)tetrahydro-2*H*-pyran-2-yl)amino)butyl)amino)tetrahydro-2*H*-pyran-3,4,5-triol) was obtained by reaction of D-glucose (36 g, 0.2 mol) with tetramethylenediamine (8.8 g, 0.1 mol) in methanol (200 mL). Reagents were stirred at room temperature for 8 h. The precipitate was filtered off under reduced pressure and dried over P_4_O_10_. Yield 95%, 78.3 g. **Mp** 108-110^o^C. **^1^H NMR** (D_2_O) δ (ppm) 1.53 (m, 4H); 2.69, 2.87 (m, m, 4H); 3.22 (m, 2H); 3.38 (m, 4H); 3.50 (m, 2H); 3.73 (m, 2H); 3.89 (m, 2H); 4.00 (d, *J* = 8,8 Hz, 2H); **^13^C NMR** (D_2_O) δ (ppm) 29.5; 47.6; 63.7; 72.7; 75.8; 79.6; 79.7; 92.4. **FTIR** ν_max_/cm^-1^ 3390 (OH, NH), 2912 and 2862 (CH), 1083 (CO).

**Hexamethylene-1,6-bis(*N*–D-glucopyranosylamine) (I-2)** (Figure S1) (IUPAC name: (2S,3R,4R,5S,6S)-2-(hydroxymethyl)-6-((6-(((2R,3R,4S,5S,6R)-3,4,5-trihydroxy-6-(hydroxymethyl)tetrahydro-2*H*-pyran-2-yl)amino)hexyl)amino)tetrahydro-2*H*-pyran-3,4,5-triol) was obtained by condensation of D-glucose (36 g, 0.2 mol) with hexamethylenediamine (11.6 g, 0.1 mol) in methanol (200 mL). Reaction mixture was stirred at room temperature for 10 h. The precipitate was filtered off under a reduced pressure and dried over P_4_O_10_. Yield 95%, 83.6 g. **Mp** 114-115^o^C. **^1^H NMR** (D_2_O) δ (ppm) 1.35 (m, 4H); 1.50 (m, 4H); 2.67, 2.87 (m, m, 4H); 3.21 (m, 2H); 3.37 (m, 4H); 3.49 (m, 2H); 3.71 (m, 2H); 3.89 (m, 2H); 3.99 (d, *J* = 8,8 Hz, 2H); **^13^C NMR** (D_2_O) δ (ppm) 28.1; 31.6; 47.8; 63.7; 72.7; 75.8; 79.5; 79.6; 92.4. **FTIR** ν_max_/cm^-1^ 3394 (OH, NH), 2930 and 2859 (CH), 1096 (CO).

**Tetramethylene-1,4-bis(*N*-deoxy-D-glucitolammonium chloride) (I-3)** (Figure S2) (IUPAC name: (2S,3R,4R,5R)-(2,3,4,5,6-pentahydroxyhexyl)({4-[(2S,3R,4R,5R)-(2,3,4,5,6-pentahydroxyhexyl)-azaniumyl]butyl}azanium dichloride) was obtained by the reaction of tetramethylene-1,4-bis(*N*–D-glucopyranosylamine) **(I-1)** (8.2 g, 0.02 mol) with sodium borohydride (1.9 g, 0.05 mol) in methanol (150 mL) at 0^o^C. The mixture was stirred at room temperature until hydrogen evolution ceased and then concentrated hydrochloric acid was added to pH of 2-3. After 24 h the crude product was filtered off under reduced pressure, dried over P_4_O_10_ and purified by crystallization from methanol:water 2:1. Yield 90%, 8.7 g. **Mp** 198-201^o^C. **^1^H NMR** (D_2_O) δ (ppm) 1.83 (m, 4H); 3.18 (m, 4H); 3.19, 3.27 (m, m, 4H); 3.66 (m, 2H); 3.68, 3.82 (m, m, 4H); 3.77 (m, 2H); 3.84 (m, 2H); 4.13 (m, 2H); **^13^C NMR** (D_2_O) δ (ppm) 25.4; 49.8; 52.3; 65.5; 71.0; 73.3; 73.4; 73.7, **FTIR** ν_max_/cm^-1^ 3410 (OH, NH), 2992 and 2885 (CH), 1093 (CO). **Elemental analysis** calculated for C_16_H_38_N_2_O_10_Cl_2_, C 39.26, H 7.77, N 5.73 % (found: C 39.19, H 7.89, N 5.69%).

**Hexamethylene-1,6-bis(*N*-deoxy-D-glucitolammonium chloride) (I-4)** (Figure S2) (IUPAC name: (2S,3R,4R,5R)-(2,3,4,5,6-pentahydroxyhexyl)({6-[(2S,3R,4R,5R)-2,3,4,5,6-pentahydroxyhexyl)-azaniumyl]hexyl}azanium dichloride) was obtained by the reaction of hexamethylene-1,6-bis(*N*–D-glucopyranosylamine) **(I-2**) (8.8 g, 0.02 mol) with sodium borohydride (1.9 g, 0.05 mol) in methanol (150 mL) at 0^o^C. The mixture was stirred at room temperature until hydrogen evolution ceased and then concentrated hydrochloric acid was added to pH of 2-3. After 24 h the crude product was filtered off under reduced pressure, dried over P_4_O_10_, and purified by crystallization from methanol:water 2:1. Yield 87%, 9.0 g. **Mp** 196-199^o^C. **^1^H NMR** (D_2_O) δ (ppm) 1.44 (m, 4H); 1.76 (m, 4H); 3.12 (m, 4H); 3.18, 3.27 (m, m, 4H); 3.65 (m, 2H); 3.67, 3.82 (m, m 4H); 3.76 (m, 2H); 3.84 (m, 2H); 4.11 (m, 2H); **^13^C NMR** (D_2_O) δ (ppm) 27.8; 28.0; 50.3; 52.1; 65.4; 70.9; 73.2; 73.3; 73.6, **FTIR** ν_max_/cm^-1^ 3417 (OH, NH), 2993 and 2880 (CH), 1095 (CO). **Elemental analysis** calculated for C_18_H_42_N_2_O_10_Cl_2_, C 41.78, H 8.12, N 5.42% (found: C 41.35, H 7.98, N 5.38%).

**Tetramethylene-1,4-bis(*N*-alkyl-*N*-deoxy-D-glucitolammonium acetates)** **(I-5 ÷ I-8)** (Figure S3) (IUPAC name: alkyl({4-[alkyl-(2S,3R,4R,5R)-(2,3,4,5,6-pentahydroxyhexyl)azaniumyl]butyl})(2S,3R,4R,5R)-(2,3,4,5,6-pentahydroxyhexyl)azane bis(acetates)): To the solution of tetramethylene-1,4-bis(*N*-deoxy-D-glucitolammonium chloride) **(I-3)** (4.89 g, 0.01 mol) in methanol (100 mL), sodium hydroxide (0.8 g, 0.02 mol) was added. The reaction mixtures were stirred, and respectively aldehyde (hexanal (2.44 mL, 0.02 mol), oktanal (3.16 mL, 0.02 mol), decanal (3.83 mL, 0.02 mol), or dodecanal (4.49 mL, 0.02 mol)) and sodium cyanoborohydride (1.65 g, 0.02 mol) were added. After 15 minutes the pH was adjusted with acetic acid (to between 6 and 8). The reaction mixtures were stirred for 18 hours at room temperature. After that time undissolved precipitates were filtered off, the solvent was evaporated and the high viscosity residues were heated in 2-propanol for 30 minutes. Undissolved precipitates were filtered off, the solvent was evaporated and the residues were washed with diethyl ether. The white products were dried over P_4_O_10_.

**Tetramethylene-1,4-bis(*N*-hexyl-*N*-deoxy-D-glucitolammonium acetate) (I-5)** (Figure S3) Yield 30 %, 2.1 g. **Mp** 110-115^o^C. **^1^H NMR** (TFA-d) δ (ppm) 0.96 (t, *J* = 6.3 Hz, 6H); 1.40 (m, 12H); 1.86 (m, 4H); 2.06 (m, 4H); 2.28 (s, 6H); 3.41 (m, 4H); 3.60 (m, 4H); 3.72 (m, 4H); 4.24 (m, 2H); 4.28, 4.41 (m, m, 4H); 4,49 (m, 2H); 4.65 (m, 2H); 4.83 (m, 2H); 7.35 (s, 2H). **^13^C NMR** (TFA-d) δ (ppm) 12.6; 19.2; 22.3; 23.9; 26.0; 31.2; 53.1; 54.6; 56.7; 62.9; 68.2; 69.7; 72.0; 74.1; 181.6. **FTIR** ν_max_/cm^-1^ 3383 (OH, NH), 2933 and 2860 (CH), 1578 (C=O), 1085 (CO). **ESIMS** m/z 293 ([M/2]^+^, 100%). **Elemental analysis** calculated for C_32_H_68_N_2_O_14_, C 54.53, H 9.72, N 3.97% (found: C 54.14, H 9.45, N 4.09%).

**Tetramethylene-1,4-bis(*N*-octyl-*N*-deoxy-D-glucitolammonium acetate) (I-6)** (Figure S3) Yield 60 %, 4.7 g. **Mp** 120-124^o^C. **^1^H NMR** (TFA-d) δ (ppm) 0.93 (t, *J* = 6.6 Hz, 6H); 1.40 (m, 20H); 1.86 (m, 4H); 2.06 (m, 4H); 2.28 (s, 6H); 3.44 (m, 4H); 3.60 (m, 4H); 3.71 (m, 4H); 4.23 (m, 2H); 4.28, 4.46(m, m, 4H); 4,50 (m, 2H); 4.67 (m, 2H); 4.82 (m, 2H); 7.35 (s, 2H). **^13^C NMR** (TFA-d) δ (ppm) 12.6; 19.0; 22.3; 23.9; 26.3; 28.9; 31.6; 53.0; 54.9; 56.6; 64.7; 68.0; 69.6; 70.3; 73.9; 181.5. **FTIR** ν_max_/cm^-1^ 3428(OH, NH), 2928 and 2857 (CH), 1571 (C=O), 1087 (CO). **ESIMS** m/z 321 ([M/2]^+^, 100%). **Elemental analysis** calculated for C_36_H_76_N_2_O_14_xH_2_O, C 55.50, H 10.09, N 3.60% (found: C 55.37, H 9.69, N 3.82%).

**Tetramethylene-1,4-bis(*N*-decyl-*N*-deoxy-D-glucitolammonium acetate) (I-7)** (Figure S3) Yield 65 %, 5.3 g. **Mp** 137-140^o^C. **^1^H NMR** (TFA-d) δ (ppm) 0.92 (t, *J* = 6.8 Hz, 6H); 1.39 (m, 28H); 1.87 (m, 4H); 2.06 (m, 4H); 2.28 (s, 6H); 3.43 (m, 4H); 3.59 (m, 4H); 3.70 (m, 4H); 4.22 (m, 2H); 4.28, 4.41 (m, m, 4H); 4,48 (m, 2H); 4.65 (m, 2H); 4.82 (m, 2H); 7.34 (s, 2H). **^13^C NMR** (TFA-d) δ (ppm) 12.9; 19.2; 22.6; 24.0; 26.3; 29.0; 29.4; 31.9; 53.2; 54.8; 56.6; 62.8; 68.2; 69.6; 72.0; 73.9; 181.6. **FTIR** ν_max_/cm^-1^ 3361 (OH, NH), 2926 and 2855 (CH), 1578 (C=O), 1083 (CO). **ESIMS** m/z 349 ([M/2]^+^, 100%). **Elemental analysis** calculated for C_40_H_84_N_2_O_14_, C 58.80, H 10.36, N 3.43% (found: C 58.98, H 10.08, N 3.81%).

**Tetramethylene-1,4-bis(*N*-dodecyl-*N*-deoxy-D-glucitolammonium acetate) (I-8)** (Figure S3) Yield 70 %, 6.2 g. **Mp** 149-153^o^C. **^1^H NMR** (TFA-d) δ (ppm) 0.91 (t, *J* = 6.6 Hz, 6H); 1.40 (m, 36H); 1.86 (m, 4H); 2.05 (m, 4H); 2.28 (s, 6H); 3.43 (m, 4H); 3.58 (m, 4H); 3.70 (m, 4H); 4.22 (m, 2H); 4.28, 4.41 (m, m, 4H); 4,47 (m, 2H); 4.65 (m, 2H); 4.82 (m, 2H); 7.36 (s, 2H). **^13^C NMR** (TFA-d) δ (ppm) 13.0; 19.2; 22.6; 24.1; 26.5; 29.5; 29.7; 32.1; 53.3; 55.0; 56.8; 64.5; 68.3; 69.7; 73.1; 74.2; 181.5. **FTIR** ν_max_/cm^-1^ 3432 (OH, NH), 2925 and 2854 (CH), 1575 (C=O), 1088 (CO). **ESIMS** m/z 377 ([M/2]^+^, 100%). **Elemental analysis** calculated for C_44_H_92_N_2_O_14_xH_2_O, C 59.30, H 10.63, N 3.14% (found: C 58.63, H 10.22, N 3.12%).

**Hexamethylene-1,6-bis(*N*-alkyl-*N*-deoxy-D-glucitolammonium acetates)** **(I-9 ÷ I-12)** (Figure S4) (IUPAC name: alkyl({6-[alkyl(2S,3R,4R,5R)-(2,3,4,5,6-pentahydroxyhexyl)azaniumyl]hexyl})(2S,3R,4R,5R)-(2,3,4,5,6-pentahydroxyhexyl)azane bis(acetates)) were obtained in the same way as products with tetramethylene spacer **(I-5 ÷ I-8)**, but to the reaction mixtures was added hexamethylene-1,6-bis(*N*-deoxy-D-glucitolammonium chloride) **(I-4)** (5.17 g, 0.01 mol).

**Hexamethylene-1,6-bis(*N*-hexyl-*N*-deoxy-D-glucitolammonium acetate)** **(I-9)** (Figure S4) Yield 20 %, 1.5 g. **Mp** 118-122^o^C. **^1^H NMR** (TFA-d) δ (ppm) 0.96 (t, *J* = 6.5 Hz, 6H); 1.42 (m, 12H); 1.57 (m, 4H) 1.89 (m, 8H); 2.28 (s, 6H); 3.39 (m, 4H); 3.55 (m, 4H); 3.67 (m, 4H); 4.23 (m, 2H); 4.28, 4.31 (m, m, 4H); 4,49 (m, 2H); 4.67 (m, 2H); 4.83 (m, 2H); 7.23 (s, 2H). **^13^C NMR** (TFA-d) δ (ppm) 11.9; 18.5; 23.2; 23.4; 25.4; 30.5; 53.1; 54.4; 55.3; 64.2; 67.5; 69.0; 71.6; 73.4; 180.8. **FTIR** ν_max_/cm^-1^ 3428 (OH, NH), 2956 and 2860 (CH), 1567 (C=O), 1084 (CO). **ESIMS** m/z 307 ([M/2]^+^, 100%). **Elemental analysis** calculated for C_34_H_72_N_2_O_14_xH_2_O, C 54.38, H 9.93, N 3.73% (found: C 54.23, H 9.99, N 3.81%).

**Hexamethylene-1,6-bis(*N*-octyl-*N*-deoxy-D-glucitolammonium acetate) (I-10)** (Figure S4) Yield 60 %, 4.7 g. **Mp** 143-146^o^C. **^1^H NMR** (TFA-d) δ (ppm) 0.92 (t, *J* = 6.8 Hz, 6H); 1.42 (m, 20H); 1.56 (m, 4H) 1.90 (m, 8H); 2.27 (s, 6H); 3.38 (m, 4H); 3.55 (m, 4H); 3.66 (m, 4H); 4.23 (m, 2H); 4.28, 4.31 (m, m, 4H); 4,47 (m, 2H); 4.67 (m, 2H); 4.83 (m, 2H); 7.23 (s, 2H). **^13^C NMR** (TFA-d) δ (ppm) 12.7; 19.1; 22.5; 23.8; 26.3; 29.0; 31.7; 53.8; 55.3; 56.3; 64.9; 68.2; 68.8; 69.6; 73.7; 181.6. **FTIR** ν_max_/cm^-1^ 3422 (OH, NH), 2955 and 2857 (CH), 1578 (C=O), 1085 (CO). **ESIMS** m/z 335 ([M/2]^+^, 100%). **Elemental analysis** calculated for C_38_H_80_N_2_O_14_, C 57.84, H 10.22, N 3.55% (found: C 58.03, H 10.09, N 3.69%).

**Hexamethylene-1,6-bis(*N*-decyl-*N*-deoxy-D-glucitolammonium acetate) (I-11)** (Figure S4) Yield 63 %, 5.3 g. **Mp** 148-153^o^C. **^1^H NMR** (TFA-d) δ (ppm) 0.92 (t, *J* = 6.9 Hz, 6H); 1.37 (m, 28H); 1.57 (m, 4H) 1.90 (m, 8H); 2.28 (s, 6H); 3.39 (m, 4H); 3.54 (m, 4H); 3.67 (m, 4H); 4.23 (m, 2H); 4.29, 4.32 (m, m, 4H); 4,49 (m, 2H); 4.66 (m, 2H); 4.82 (m, 2H); 7.18 (s, 2H). **^13^C NMR** (TFA-d) δ (ppm) 12.9; 19.1; 22.5; 23.9; 26.3; 29.3; 29.4; 31.9; 53.7; 55.2; 56.1; 65.0; 68.3; 68.9; 72.3; 74.1; 181.6. **FTIR** ν_max_/cm^-1^ 3437 (OH, NH), 2958 and 2855 (CH), 1578 (C=O), 1050 (CO). **ESIMS** m/z 363 ([M/2]^+^, 100%). **Elemental analysis** calculated for C_42_H_88_N_2_O_14_, C 59.69, H 10.49, N 3.31% (found: C 59.83, H 10.30, N 3.37%).

**Hexamethylene-1,6-bis(*N*-dodecyl-*N*-deoxy-D-glucitolammonium acetate) (I-12)** (Figure S4) Yield 68 %, 6.1 g. **Mp** 155-160^o^C. **^1^H NMR** (TFA-d) δ (ppm) 0.91 (t, *J* = 5.9 Hz, 6H); 1.40 (m, 36H); 1.57 (m, 4H) 1.89 (m, 8H); 2.28 (s, 6H); 3.39 (m, 4H); 3.57 (m, 4H); 3.68 (m, 4H); 4.23 (m, 2H); 4.29, 4.30 (m, m, 4H); 4,49 (m, 2H); 4.66 (m, 2H); 4.84 (m, 2H); 7.14 (s, 2H). **^13^C NMR** (TFA-d) δ (ppm) 12.8; 19.1; 22.5; 23.9; 26.3; 29.3; 29.6; 31.9; 53.8; 55.3; 56.1; 62.8; 68.8; 69.6; 70.7; 73.9; 181.5. **FTIR** ν_max_/cm^-1^ 3427 (OH, NH), 2956 and 2853 (CH), 1575 (C=O), 1061 (CO). **ESIMS** m/z 391 ([M/2]^+^, 100%). **Elemental analysis** calculated for C_46_H_96_N_2_O_14_, C 61.30, H 10.74, N 3.11% (found: C 61.46, H 10.48, N 3.16%).

**Tetramethylene-1,4-bis(*N*-dodecyl-*N*-deoxy-D-glucitolamine) (I-13)** (Figure S5)

(IUPAC name: (2R,2’R,3R,3’R,4R,4’R,5S,5’S)-6,6’-butane-1,4-diylbis(dodecylazanediyl)bis (hexane-1,2,3,4,5-pentaol) was obtained in the reaction of tetramethylene-1,4-bis(*N*-dodecyl-*N*-deoxy-D-glucitolammonium acetate) **(I-8)** (8.73 g, 0.01 mol) with sodium hydroxide (1.4 g, 0.025 mol) in methanol (50 mL). Reaction was carried out on the magnetic stirrer in one hour. Then product was filtered out and dried. Yield 95%, 7.15 g. **Mp** 290-293^o^C. **FTIR** ν_max_/cm^-1^ 3382 (OH), 2925 and 2854 (CH), 1099 (CO).

**Tetramethylene-1,4-bis(*N*-alkyl-*N*-octyl-*N*-deoxy-D-glucitolammonium iodides) (1÷3)** (Figure S6) (IUPAC name: alkyl({4-[alkyl(octyl)-(2S,3R,4R,5R)-(2,3,4,5,6-pentahydroxyhexyl) azaniumyl]-butyl})octyl-(2S,3R,4R,5R)-(2,3,4,5,6-pentahydroxyhexyl)azane bis(iodides)): Monohydrated tetramethylene-1,4-bis(*N*-octyl-*N*-deoxy-D-glucitolammonium acetate) **(I-6)** (7.79 g, 0.01 mol) was dissolved in *N,N*-dimethylformamide (50 mL). Then potassium hydroxide (1.12 g, 0.12 mol) and aliphatic iodide (iodoethane (2 mL, 0.025 mol), 1-iodopropane (2.4 mL, 0.025 mol) or 1-iodododecan (6.16 mL, 0.025 mol) respectively) were added. The reaction mixtures were heated in 152^o^C for 24 h. After that time solvent was evaporated, and residue was washed with diethyl ether. The white precipitates were filtered under vacuum, dried over P_4_O_10_ and purified by three times crystallization from nitromethane:methanol 3:2.

**Tetramethylene-1,4-bis(*N*-ethyl-*N*-octyl-*N*-deoxy-D-glucitolammonium iodide)** **(1)** (Figure S6) Yield 40 %, 3.8 g. **Mp** 230^o^C (decomp.). **^1^H NMR** (D_2_O) δ (ppm) 0.89 (m, 6H); 1.31 (m, 20H); 1.60 (m, 6H); 1.80 (m, 4H); 2.18 (m, 4H); 3.35-3.76 (m, 16H); 4.31-4.88 (m, 12H). **FTIR** ν_max_/cm^-1^ 3367 (OH), 2927 and 2857 (CH), 965 (CO). **Elemental analysis** calculated for C_36_H_78_N_2_O_10_I_2_, C 45.38, H 8.25, N 2.94% (found: C 45.61, H 7.99, N 3.09%).

**Tetramethylene-1,4-bis(*N*-propyl-*N*-octyl-*N*-deoxy-D-glucitolammonium iodide) (2)** (Figure S6) Yield 45 %, 4.4 g. **Mp** 235^o^C (decomp.). **^1^H NMR** (D_2_O) δ (ppm) 0.87 (m, 6H); 1.30 (m, 24H); 1.60 (m, 6H); 1.87 (m, 4H); 2.19 (m, 4H); 3.39-3.76 (m, 16H); 4.08-4.80 (m, 12H). **FTIR** ν_max_/cm^-1^ 3379 (OH), 2925 and 2855 (CH), 963 (CO). **Elemental analysis** calculated for C_38_H_82_N_2_O_10_I_2_, C 46.53, H 8.43, N 2.86% (found: C 46.81, H 8.03, N 2.98%).

**Tetramethylene-1,4-bis(*N*-dodecyl-*N*-octyl-*N*-deoxy-D-glucitolammonium iodide)** **(3)** (Figure S6) Yield 65 %, 8.0 g. **Mp** 250^o^C (decomp.). **^1^H NMR** (TFA-d) δ (ppm) 0.91 (m, 12H); 1.35 (m, 56H); 1.86 (m, 8H); 2.21 (m, 4H); 3.45 (m, 8H); 3.81 (m, 4H); 3.94 (m, 4H); 4.10-4.83 (m, 12H). **FTIR** ν_max_/cm^-1^ 3434 (OH), 2930 and 2860 (CH), 962 (CO). **Elemental analysis** calculated for C_56_H_118_N_2_O_10_I_2_, C 54.53, H 9.64, N 2.27% (found: C 54.87, H 9.48, N 2.54%).

**Tetramethylene-1,4-bis(*N*-alkyl-*N*-decyl-*N*-deoxy-D-glucitolammonium iodides) (4÷6)** (Figure S7) (IUPAC name: Alkyl({4-[alkyl(decyl)-(2S,3R,4R,5R)-(2,3,4,5,6-pentahydroxyhexyl) azaniumyl]-butyl})decyl-(2S,3R,4R,5R)-(2,3,4,5,6-pentahydroxyhexyl)azane bis(iodides)) were obtained in the same way as tetramethylene-1,4-bis(*N*-alkyl-*N*-octyl*-N*-deoxy-D-glucitolammonium iodides) **(1÷3)**, but to the reaction mixtures was added tetramethylene-1,4-bis(*N*-decyl-*N*-deoxy-D-glucitolammonium acetate) **(I-7)** (8.17 g, 0.01 mol).

**Tetramethylene-1,4-bis(*N*-ethyl*-N*-decyl-*N*-deoxy-D-glucitolammonium iodide) (4)** (Figure S7) Yield 35 %, 3.5 g. **Mp** 240^o^C (decomp.). **^1^H NMR** (TFA-d) δ (ppm) 0.91 (m, 6H); 1.35 (m, 34H); 1.83 (m, 4H); 2.19 (m, 4H); 3.42, 3.66 (m, m, 8H); 3.74 (m, 4H); 3.92 (m, 4H); 4.10-4.99 (m, 12H). **FTIR** ν_max_/cm^-1^ 3381 (OH), 2925 and 2854 (CH), 963 (CO). **Elemental analysis** calculated for C_40_H_86_N_2_O_10_I_2_, C 47.62, H 8.59, N 2.78% (found: C 48.06, H 8.26, N 2.66%).

**Tetramethylene-1,4-bis(*N*-propyl-*N*-decyl-*N*-deoxy-D-glucitolammonium iodide) (5)** (Figure S7) Yield 40 %, 4.1 g. **Mp** 243^o^C (decomp.). **^1^H NMR** (TFA-d) δ (ppm) 0.92 (m, 6H); 1.36 (m, 34H); 1.86 (m, 4H); 2.12 (m, 4H); 2.41 (m, 4H); 3.44, 3.54 (m, m, 8H); 3.77 (m, 4H); 3.93 (m, 4H); 4.10-4.85 (m, 12H). **FTIR** ν_max_/cm^-1^ 3372 (OH), 2925 and 2854 (CH), 963 (CO). **Elemental analysis** calculated for C_42_H_90_N_2_O_10_I_2_, C 48.65, H 8.75, N 2.70% (found: C 48.86, H 8.28, N 2.91%).

**Tetramethylene-1,4-bis(*N*-dodecyl-*N*-decyl-*N*-deoxy-D-glucitolammonium iodide) (6)** (Figure S7) Yield 65 %, 8.4 g. **Mp** 255^o^C (decomp.). **^1^H NMR** (TFA-d) δ (ppm) 0.91 (m, 12H); 1.35 (m, 64H); 1.83 (m, 8H); 2.18 (m, 4H); 3.40 (m, 8H); 3.73 (m, 4H); 3.92 (m, 4H); 4.10-4.83 (m, 12H). **FTIR** ν_max_/cm^-1^ 3368 (OH), 2924 and 2853 (CH), 962 (CO). **Elemental analysis** calculated for C_60_H_126_N_2_O_10_I_2_, 55.89, H 9.85, N 2.17% (found: C 56.08, H 9.59, N 2.56%).

**Tetramethylene-1,4-bis(*N*-alkyl-*N*-dodecyl-*N*-deoxy-D-glucitolammonium iodides)** **(7÷9)** (Figure S8) (IUPAC name: Alkyl({4-[alkyl(dodecyl)-(2S,3R,4R,5R)-(2,3,4,5,6-pentahydroxyhexyl)azaniumyl]butyl})-dodecyl-(2S,3R,4R,5R)-(2,3,4,5,6-pentahydroxyhexyl)azane bis(iodides)) were obtained in the same way as tetramethylene-1,4-bis(*N*-alkyl-*N*-octyl-*N*-deoxy-D-glucitolammonium iodides) **(1÷3)**, but to the reaction mixtures was added monohydrated tetramethylene-1,4-bis(*N*-dodecyl-*N*-deoxy-D-glucitolammonium acetate) **(I-8)** (8.91 g, 0.01 mol)

**Tetramethylene-1,4-bis(*N*-ethyl-*N*-dodecyl-*N*-deoxy-D-glucitolammonium iodide)** **(7)** (Figure S8) Yield 50 %, 5.3 g. **Mp** 235^o^C (decomp.). **^1^H NMR** (TFA-d) δ (ppm) 0.92 (m, 6H); 1.36 (m, 42H); 1.83 (m, 4H); 2.12 (m, 4H); 3.46, 3.64 (m, m, 8H); 3.83 (m, 4H); 3.89(m, 4H); 4.10-4.95 (m, 12H). **FTIR** ν_max_/cm^-1^ 3367 (OH), 2924 and 2853 (CH), 964 (CO). **Elemental analysis** calculated for C_44_H_94_N_2_O_10_I_2_, C 49.62, H 8.90, N 2.63% (found: C 50.03, H 8.63, N 2.23%).

**Tetramethylene-1,4-bis(*N*-propyl-*N*-dodecyl-*N*-deoxy-D-glucitolammonium iodide)** **(8)** (Figure S8) Yield 60 %, 6.5 g. **Mp** 240^o^C (decomp.). **^1^H NMR** (TFA-d) δ (ppm) 0.91 (m, 6H); 1.35 (m, 42H); 1.89 (m, 4H); 2.18 (m, 4H); 2.39 (m, 4H); 3.41, 3.50 (m, m, 8H); 3.83 (m, 4H); 3.91 (m, 4H); 4.10-4.97 (m, 12H). **FTIR** ν_max_/cm^-1^ 3371 (OH), 2924 and 2853 (CH), 963 (CO). **Elemental analysis** calculated for C_46_H_98_N_2_O_10_I_2_, C 50.54, H 9.04, N 2.56% (found: C 50.87, H 8.87, N 2.79%).

**Tetramethylene-1,4-bis(*N,N*-didodecyl-*N*-deoxy-D-glucitolammonium iodide)** **(9)** (Figure S8) Yield 68 %, 9.1 g. **Mp** 257^o^C (decomp.). **^1^H NMR** (TFA-d) δ (ppm) 0.91 (m, 12H); 1.35 (m, 72H); 1.89 (m, 8H); 2.12 (m, 4H); 3.49 (m, 8H); 3.64 (m, 4H); 3.89 (m, 4H); 4.10-4.84 (m, 12H). **FTIR** ν_max_/cm^-1^ 3422 (OH), 2923 and 2853 (CH), 961 (CO). **Elemental analysis** calculated for C_64_H_134_N_2_O_10_I_2_, C 57.13, H 10.04, N 2.08% (found: C 57.37, H 9.87, N 2.29%).

**Hexamethylene-1,6-bis(*N*-dodecyl-*N*-alkyl-*N*-deoxy-D-glucitolammonium iodides) (10÷12)** (Figure S9) (IUPAC name: Alkyl({6-[alkyl(dodecyl)(2S,3R,4R,5R)-(2,3,4,5,6-pentahydroxyhexyl) azaniumyl]-heksyl})-dodecyl-(2S,3R,4R,5R)-(2,3,4,5,6-pentahydroxyhexyl)azane bis(iodides)) were obtained in the same way as analogue with tetramethylene spacer **(1÷9)**, but to the reaction mixtures was added 1-iodododecan (6.16 mL, 0.025 mol) and hexamethylene-1,6-bis(*N*-octyl-*N*-deoxy-D-glucitolammonium acetate) **(I-10)** (7.89 g, 0.01 mol), hexamethylene-1,6-bis(*N*-decyl-*N*-deoxy-D-glucitolammonium acetate) (8.45 g, 0.01 mol) **(I-11)** or hexamethylene-1,6-bis(*N*-dodecyl-*N*-deoxy-D-glucitolammonium acetate) **(I-12)** (9.01 g, 0.01 mol) respectively.

**Hexamethylene-1,6-bis(*N*-dodecyl-*N*-octyl-*N*-deoxy-D-glucitolammonium iodide)** **(10)** (Figure S9) Yield 60 %, 7.6 g. **Mp** 245^o^C (decomp.). **^1^H NMR** (TFA-d) δ (ppm) 0.91 (m, 12H); 1.35 (m, 56H); 1.64 (m, 4H); 1.91 (m, 12H); 3.42 (m, 8H); 3.68 (m, 4H); 3.93 (m, 4H); 4.25-4.67 (m, 12H). **FTIR** ν_max_/cm^-1^ 3421 (OH), 2956 and 2831 (CH), 966 (CO). **Elemental analysis** calculated for C_58_H_122_N_2_O_10_I_2_, C 55.23, H 9.75, N 2.22% (found: C 55.62, H 9.55, N 2.40%).

**Hexamethylene-1,6-bis(*N*-dodecyl-*N*-decyl-*N*-deoxy-D-glucitolammonium iodide)** **(11)** (Figure S9) Yield 65 %, 8.6 g. **Mp** 269^o^C (decomp.). **^1^H NMR** (TFA-d) δ (ppm) 0.92 (m, 12H); 1.35 (m, 64H); 1.58 (m, 4H); 1.86 (m, 12H); 3.40 (m, 8H); 3.67 (m, 4H); 3.93 (m, 4H); 4.13-4.90 (m, 12H). **FTIR** ν_max_/cm^-1^ 3425 (OH), 2925 and 2854 (CH), 962 (CO). **Elemental analysis** calculated for C_62_H_130_N_2_O_10_I_2_, C 56.52, H 9.95, N 2.13% (found C 56.87, H 9.71, N 2.30%).

**Hexamethylene-1,6-bis(*N,N*-didodecyl-*N*-deoxy-D-glucitolammonium iodide)** **(12)** (Figure S9) Yield 65 %, 8.9 g. **Mp** 270^o^C (decomp.). **^1^H NMR** (TFA-d) δ (ppm) 0.91 (m, 12H); 1.35 (m, 72H); 1.56 (m, 4H); 1.86 (m, 12H); 3.40 (m, 8H); 3.67 (m, 4H); 3.90 (m, 4H); 4.25-4.82 (m, 12H). **FTIR** ν_max_/cm^-1^ 3425 (OH), 2924 and 2854 (CH), 962 (CO). **Elemental analysis** calculated for C_66_H_138_N_2_O_10_I_2_, C 57.71, H 10.13, N 2.04% (found: C 57.98, H 9.94, N 2.38%).
